# *S. pombe* placed on the prion map

**DOI:** 10.15698/mic2017.02.555

**Published:** 2017-02-03

**Authors:** Jacqueline Hayles

**Affiliations:** 1 Cell Cycle Laboratory, The Francis Crick Institute, I, Midland Road, London NW1 1AT, United Kingdom.

**Keywords:** S. pombe, Ctr4, prion, Hsp104, fission yeast

## Abstract

*Schizosaccharomyces pombe* has been used extensively as a model organism, however it is only recently that the first prion in this organism, a copper transporter protein encoded by *ctr4,* has been conclusively demonstrated. Prions are found in a wide range of organisms and have been implicated in a number of human neurodegenerative diseases. Research into the biology of prions has been carried out mainly in the budding yeast *Saccharomyces cerevisiae, *however there are many questions still to be addressed*. *Now, with the identification of the Ctr4 prion in *S. pombe,* further work in the two yeasts and comparisons of prion biology in these organisms should lead to a greater understanding of prions and their role in disease.

It is now well established that protein-based epigenetic inheritance occurs in a number of different organisms including Metazoa, plants, fungi and bacteria 1,2,3,4. The proteins responsible for this type of inheritance, known as prions, are amyloid forms of cellular proteins that usually contain asparagine (N)/glutamine (Q) rich domains. Prions have been implicated in a number of severe neurodegenerative disorders that cause Dementia in humans, particularly in older people, and which are currently incurable 5,6. As well as the personal cost to Dementia patients and their families, with an ageing population these diseases also pose a severe burden for society 7. For these reasons finding a cure is an important goal for medical research.

An understanding of the biology of prions and their role in human diseases is essential for the development of treatments. Prion biology is also an interesting biological problem in itself and there are still many questions to be addressed. For example, is there a normal biological function for prions and how does this vary across species? What is the basis of their infectivity? Much of the work investigating prion biology has been carried out in the budding yeast *Saccharomyces cerevisiae*, which is an excellent, long-standing model for eukaryotic cell biology. So far only two fungi are known to have prions: *S. cerevisiae* and *Podospora anserina *3*, *and although prion-like mechanisms have also been shown in the fission yeast* Schizosaccharomyces pombe *8*, *it is only now (Sideri *et al.* 9) that evidence for the existence of a prion in this organism has been conclusively demonstrated.

*S. pombe* is also an excellent model system for eukaryotic cell biology and indeed is often the organism of choice for certain areas of research, such as chromatin organisation 10. It is a simple rod-shaped unicellular organism that is only distantly related to *S. cerevisiae*, many genetic and molecular techniques are available, its 12.5 Mb genome is sequenced, and there has been rapid development of post-genomic resources and techniques 11. With the demonstration of a prion in *S. pombe* it is now possible to take a complementary approach to work in *S. cerevisiae* and to be able to compare and contrast prion biology in these two yeasts, which will hopefully rapidly advance our current understanding.

Sideri *et al. *show that, as in *S. cerevisiae*, overexpressed ScSup35-GFP in *S. pombe* forms aggregates that are dependent on the chaperone Hsp104 and that this prion-like state (*PSI*^+^) is transmissible. Having established that *S. pombe* contains all the endogenous cellular mechanisms required for the propagation of the budding yeast prion ScSup35, they proceeded to screen for endogenous *S. pombe* prion-like proteins. Two approaches were used: 1) a screen of 80 candidate proteins selected as insoluble and detergent-resistant or N/Q rich proteins, and 2) a screen of the proteome for proteins containing prion-forming domains, PrDs. The first screen failed to identify any proteins that were positive for each of their assays for prion-like features, and was not pursued any further. However the second approach identified 295 PrD containing proteins, including two proteins, Fib1 and Myo2, which were candidates for the first screen. Many of these 295 proteins are located at the membrane or cell surface, and from a secondary screen of 30 proteins Sideri *et al.* selected for further study the copper transporter protein, Ctr4, as a putative *S. pombe* prion.

Ctr4 contains a 55 base pair PrD consisting of ten N, but no Q residues. This region mapped to a disordered region of the protein, which is typical of PrDs in *S. cerevisae*. Overexpressed Ctr4 did not form aggregates as distinct cytoplasmic foci but, like endogenously expressed protein, localised to the plasma membrane. Here it showed evidence of clustering and formation of ribbon-like structures, whereas endogenously expressed Ctr4 was more evenly distributed in the cell membrane. Overexpressed Ctr4 was resistant to Proteinase K, heat treatment and 2% SDS, suggesting that it was in an altered conformational state. Unlike ScSup35, the prion-like features of overexpressed Ctr4 were not dependent on the SpHsp104 chaperone.

Overexpressed Ctr4 conferred sensitivity to oxidative stress, and Sideri *et al.* used this phenotype to determine whether protein extracts from cells overexpressing Ctr4 conferred hydrogen peroxide (H_2_O_2_) sensitivity to wild type cells. They found that indeed several transformants of wild type cells had acquired H_2_O_2_ sensitivity. Furthermore this transmissible activity showed non-Mendelian segregation. Taken together, the prion-like characteristics of overexpressed Ctr4 confirm that it is the first *bona fide* prion in *S. pombe* and opens up a new area of research in this organism.

So, what is the future of prion research in fission yeast and the wider field? There are many outstanding questions and mentioned here are just a few of immediate interest for comparison between the two yeasts. Already some differences in prion biology have emerged; for example, Sideri *et al.* have shown that Ctr4 is independent of Hsp104, unlike *S. cerevisiae* prions where the Hsp104/70/40 chaperone machinery is thought to play a central role in prion propagation 12,13. Does a different chaperone machinery carry out this role in *S. pombe*, as Sideri *et al. *suggest, or is it possible that in some circumstances prion propagation is chaperone independent? As yet there is no known orthologue of Hsp104 in mammals, although Hsp110 is thought to fulfil this role 14. What is the significance of this difference in chaperone machinery, does it have any effect on prion toxicity or transmission?

There is also a difference between the percentage of N and Q rich domains in the *S. cerevisiae* proteome (2.7%) compared to humans (0.9%) and *S. pombe* (0.4%) 15. Why is there such a difference? A tendency to form N-rich proteins arose early during *Saccharomycetes* evolution and it has been suggested that this may have contributed to the increased number of prions in *S. cerevisiae* 16. Are* S. pombe* (and human) amyloid proteins more toxic than those of *S*. *cerevisiae*? And did this potential toxicity lead to some loss of PrDs during evolution for *Schizosaccharomycetes*, in a similar way to that proposed for the *Eurotiales* clade? It will be of interest to compare PrDs between *S. cerevisiae *and* S. pombe* for any relevance to the biology of prions in these organisms.

It has also been shown that *S. cerevisiae* prion-like behaviour can lead to increased diversity which confers a selective advantage in certain growth conditions 17. Do *S. pombe* prions also confer a selective advantage and how wide spread is this phenomenon amongst other Eukaryotes and Prokaryotes? The Bähler group and others now have a large dataset of putative prion-like proteins in fission yeast and can certainly start to address some of these questions.

Whilst much research has focussed on non-Mendelian protein-based inheritance by prions, it has been shown that in *S. cerevisiae* this can also occur in a prion-independent manner, though still with the requirement of chaperones for maintenance of the phenotype 18. This of course raises the interesting possibility that some of the putative prion candidates identified by Sideri *et al.* may fall into this category.

Though there has been a considerable advance in our understanding of protein-based inheritance, it seems that the field is constantly giving rise to the unexpected, opening up new and exciting areas of research. The two model organisms, *S. cerevisiae* and *S. pombe*, are likely to play central roles in future research, advancing our understanding of prion biology and the wider mechanism of protein-based inheritance.
